# Supplementary material for: FBXO38 is dispensable for PD-1 regulation
Source: EMBO Rep. 2024 Sep 12;25(10):13. doi: 10.1038/s44319-024-00220-8 (PMC11467412; doi:10.1038/s44319-024-00220-8)
Supplement: Supplementary file 2 — Appendix [file 44319_2024_220_MOESM2_ESM.pdf]

Appendix

Appendix Table S1..p. 1

Appendix Table S1: Approximate calculation of hPD1 mRNA copy number in HEK-293FT-pSB-hPD-1

	PD-1 cDNA copies			
**Ct values**	**1436000**	**143600**	**14360**	**1436**
**Ct value #1**	22.09	28.07	32.73	37.78
**Ct value #2**	21.94	28.25	32.55	37.26
**Ct value #3**	22.33	27.90	32.81	37.70
**Mean Ct**	22.12	28.07	32.70	37.58

Measured Ct values of PD-1 using different concentration of pSB-hPD1 plasmid

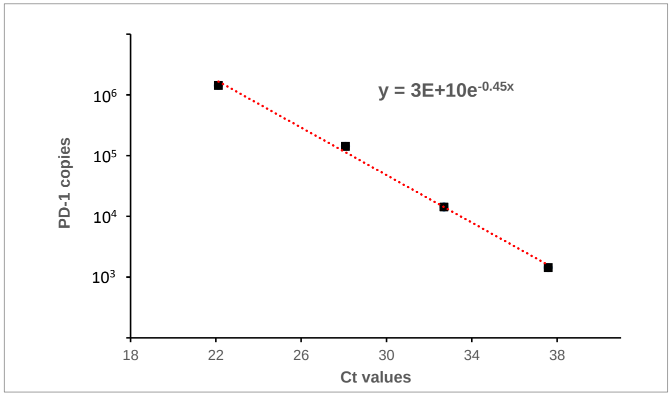

For each qPCR reaction 0.0125 µg of reverse transcribed RNA was used

	HEK293T-hPD1 (cDNA/0.0125 µg of RNA)		
**Doxycycline conc.**	**1 µg/ml**	**0.1 µg/ml**	**0.01 µg/ml**
	**RPL23A**		
**Ct value #1**	19.96	19.57	19.34
**Ct value #2**	19.91	19.75	19.46
**Ct value #3**	20	19.81	19.58
	**PD-1**		
**Ct value #1**	31.78	32.79	36.98
**Ct value #2**	31.67	33.05	36.19
**Ct value #3**	31.7	32.92	36.85

**Mean RPL23A Ct**	19.96	19.71	19.46
**Norm. factor**	0.00	-0.25	-0.50

	PD-1		
**Norm. Ct value #1**	31.78	33.04	37.48
**Norm. Ct value #2**	31.67	33.30	36.69
**Norm. Ct value #3**	31.70	33.17	37.35

**Mean PD-1 Norm. Ct**	31.72	33.17	37.17

Using y = 3E+10e-0.45x

	PD-1 cDNA copies in 0.0125 µg RNA		
**PD-1 cDNA copies**	18995.61	9891.82	1632.66

	PD-1 cDNA copies in 1 µg RNA		
**PD-1 cDNA copies**	1519648.86	791345.43	130612.45

Original yield was 0.9 µg of RNA from 10^5 cells

	PD-1 cDNA copies in 1 cell		
**Doxycycline conc.**	**1 µg/ml**	**0.1 µg/ml**	**0.01 µg/ml**
**hPD-1 cDNA copies**	**17**	**9**	**1**

Approximate calculation of hPD-1 mRNA copies per cell (HEK-293FT-pSB-hPD-1)

Comparison of results with publicly available measurement of PD1 copies in activated T-cells

Assuming that 1-4 RPKM is ~ 1 copy of RNA per cell

Djebali S et al. Nature. 2012 Sep 6;489(7414):101-8. doi: 10.1038/nature11233. PMID: 22955620; PMCID: PMC3684276.

Experiment #	max. CPM of *mPDCD1*
GSE176310	68
GSE116697	43
GSE90569	50
GSE122149	22
