# Supplementary figures and images for: FBXO38 is dispensable for PD-1 regulation
Source: EMBO Rep. 2024 Sep 12;25(10):13. doi: 10.1038/s44319-024-00220-8 (PMC11467412; doi:10.1038/s44319-024-00220-8)

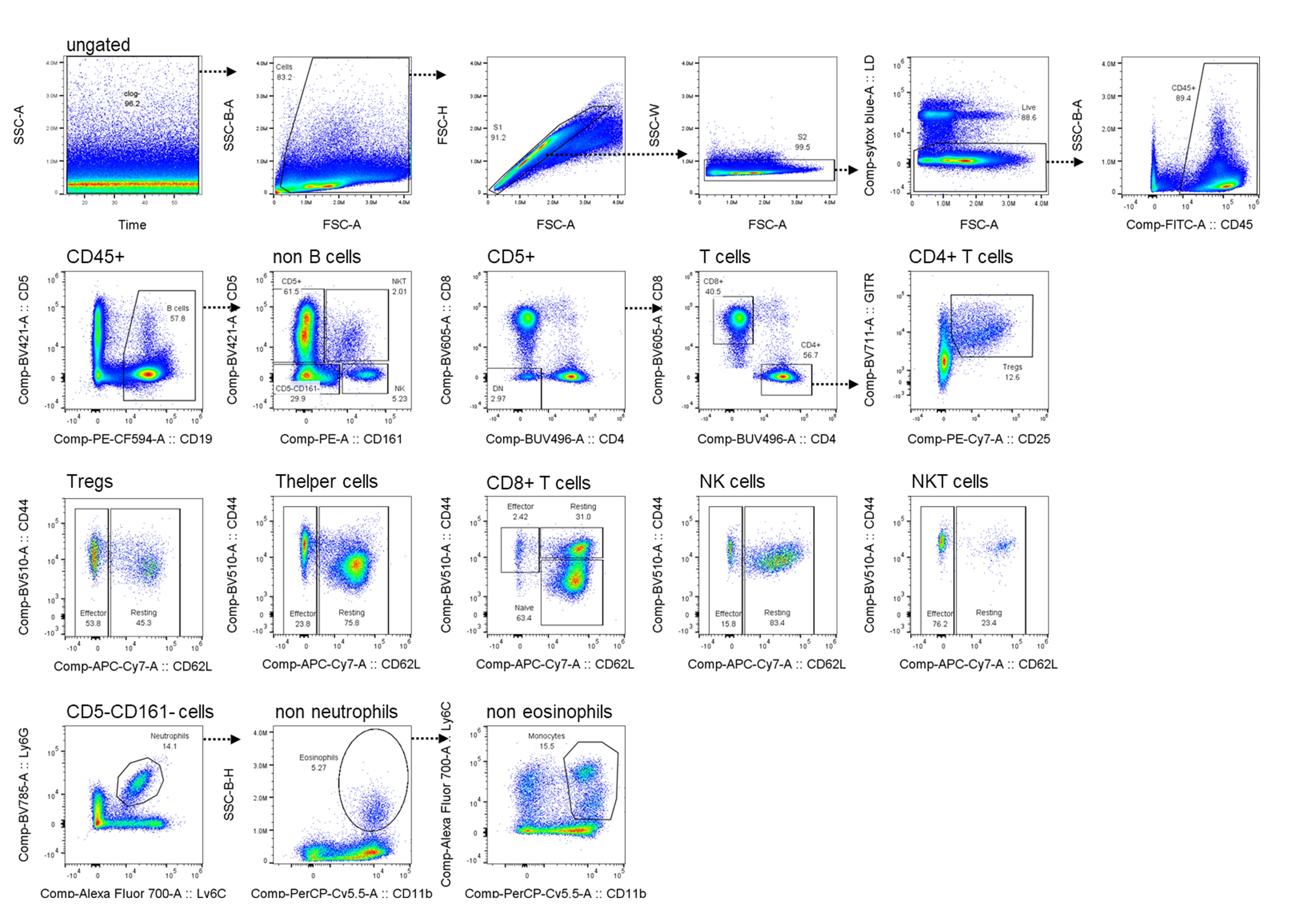

Supplement: Supplementary file 3 — Source data Fig. EV1 [file 44319_2024_220_MOESM3_ESM.zip › Expanded View Figure 1/EV1C/Gating strategy Figure EV1C.JPG]

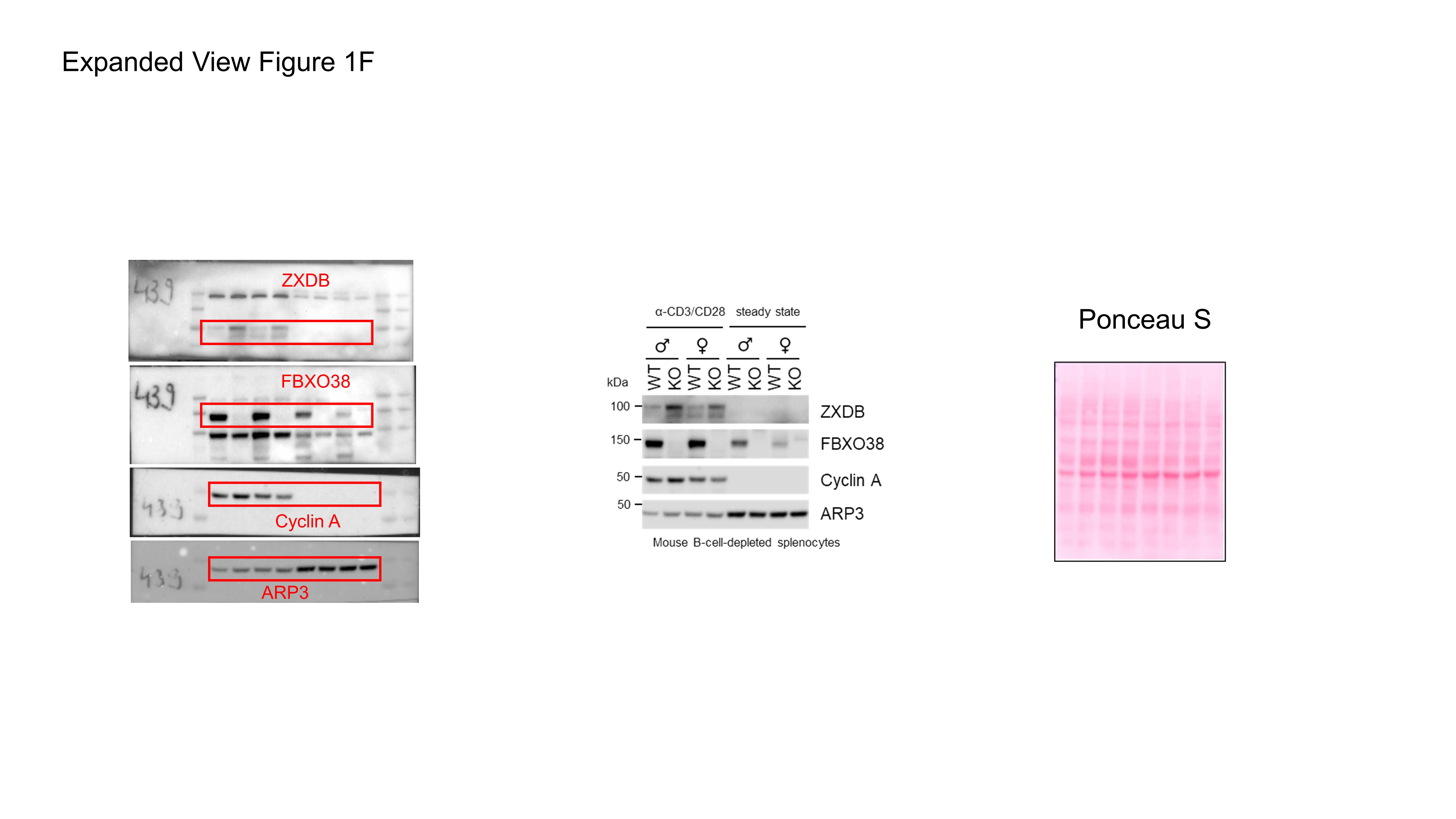

Supplement: Supplementary file 3 — Source data Fig. EV1 [file 44319_2024_220_MOESM3_ESM.zip › Expanded View Figure 1/EV1F/EV1F.JPG]

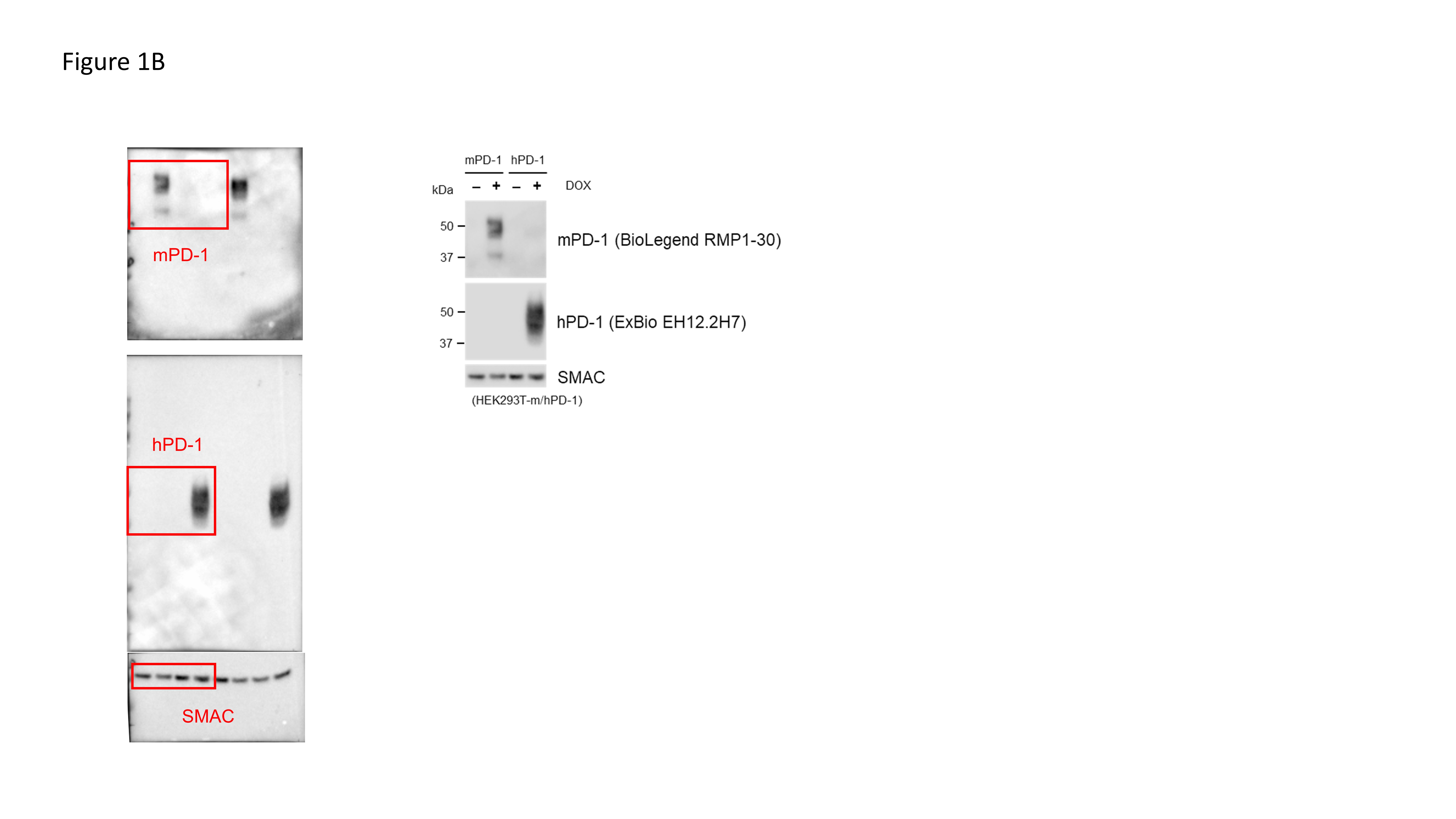

Supplement: Supplementary file 4 — Source data Fig. 1 [file 44319_2024_220_MOESM4_ESM.zip › Figure 1/FIG 1B/1B.JPG]

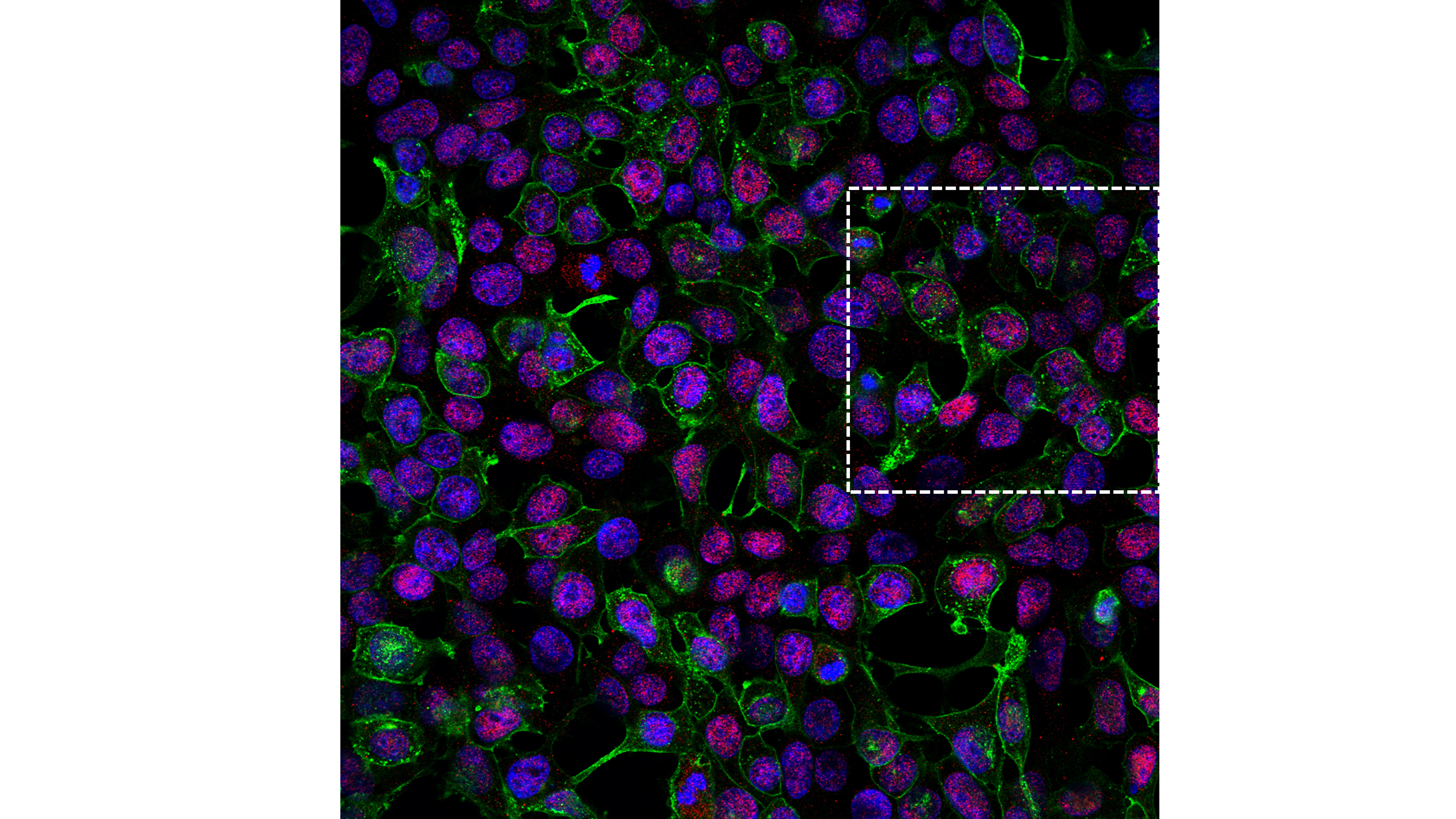

Supplement: Supplementary file 4 — Source data Fig. 1 [file 44319_2024_220_MOESM4_ESM.zip › Figure 1/FIG 1C/Fig_1C_roi/1C_subset.jpg]

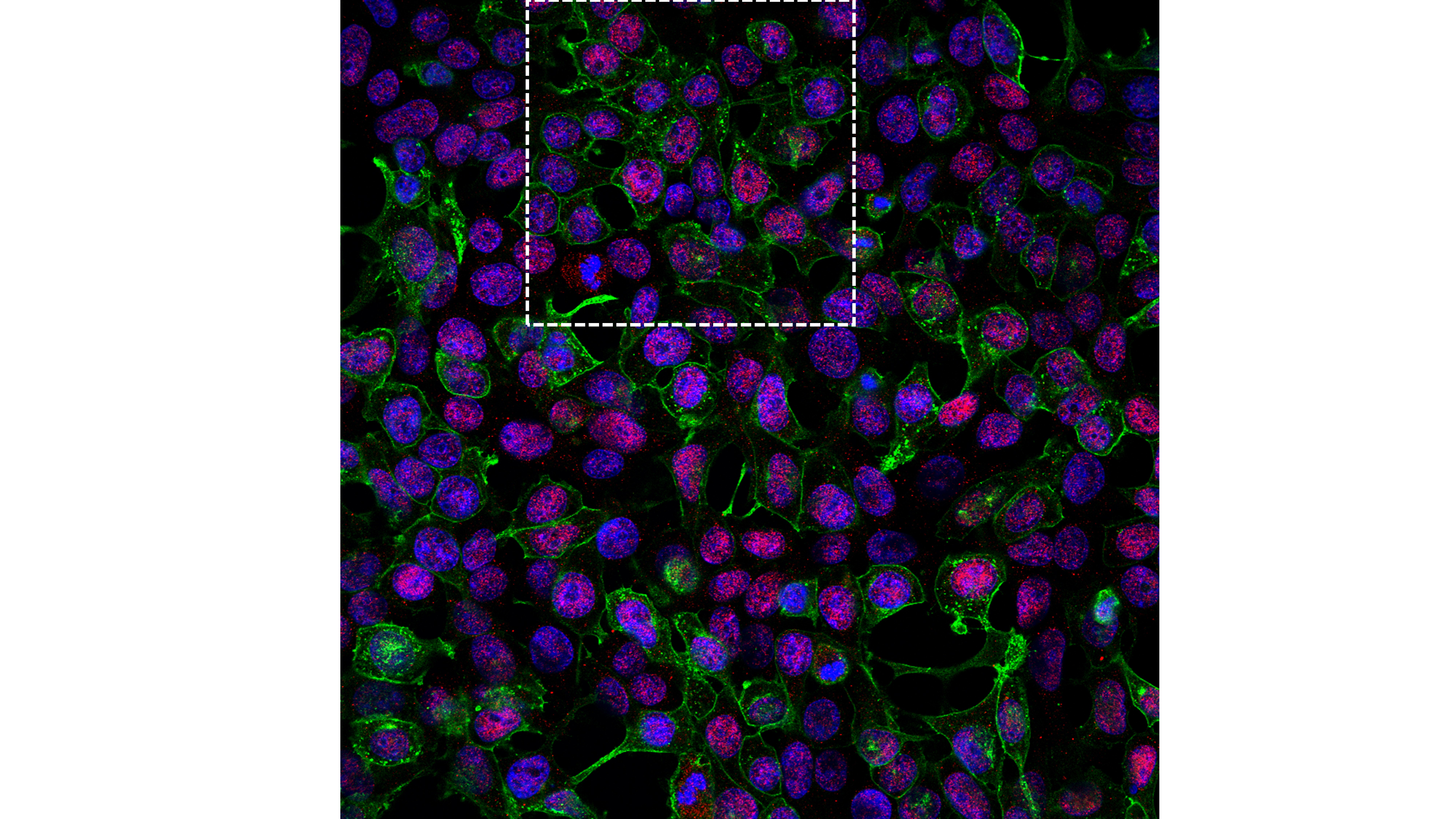

Supplement: Supplementary file 4 — Source data Fig. 1 [file 44319_2024_220_MOESM4_ESM.zip › Figure 1/FIG 1E/Fig_1E_roi/1E_DMSO_DOX_subset.JPG]

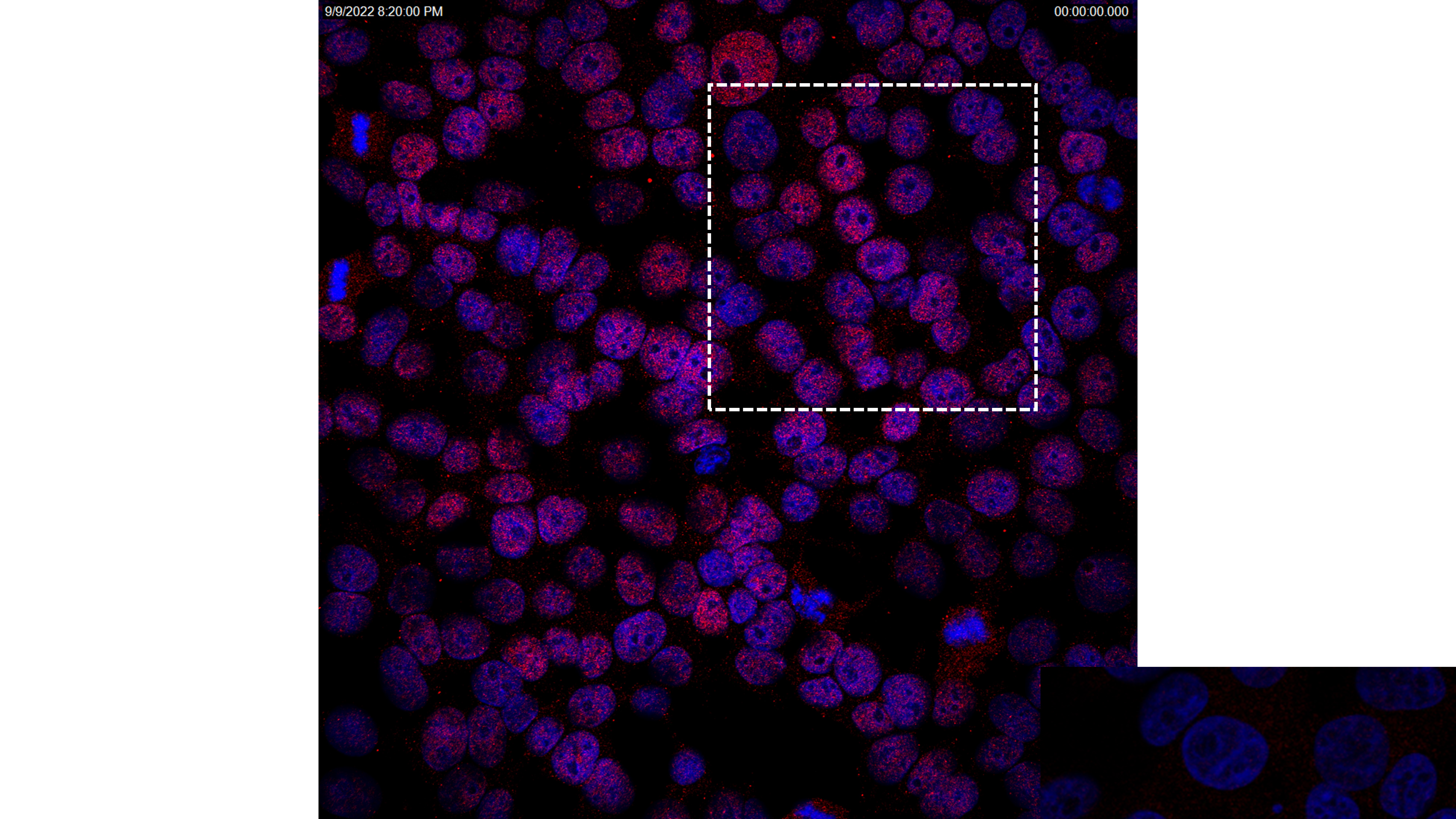

Supplement: Supplementary file 4 — Source data Fig. 1 [file 44319_2024_220_MOESM4_ESM.zip › Figure 1/FIG 1E/Fig_1E_roi/1E_DMSO_NO_DOX_subset.JPG]

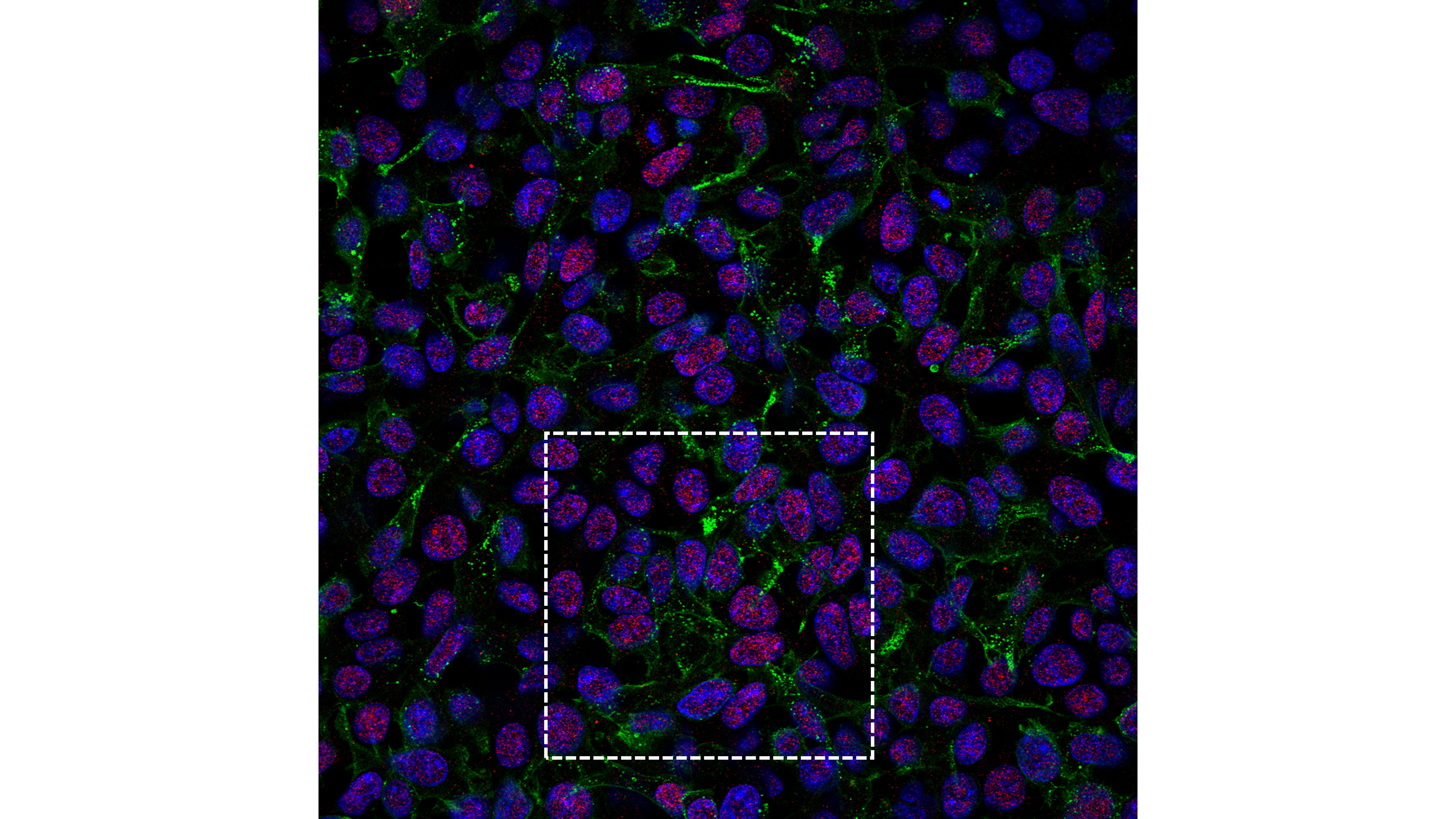

Supplement: Supplementary file 4 — Source data Fig. 1 [file 44319_2024_220_MOESM4_ESM.zip › Figure 1/FIG 1E/Fig_1E_roi/1E_MG132_DOX_subset.JPG]

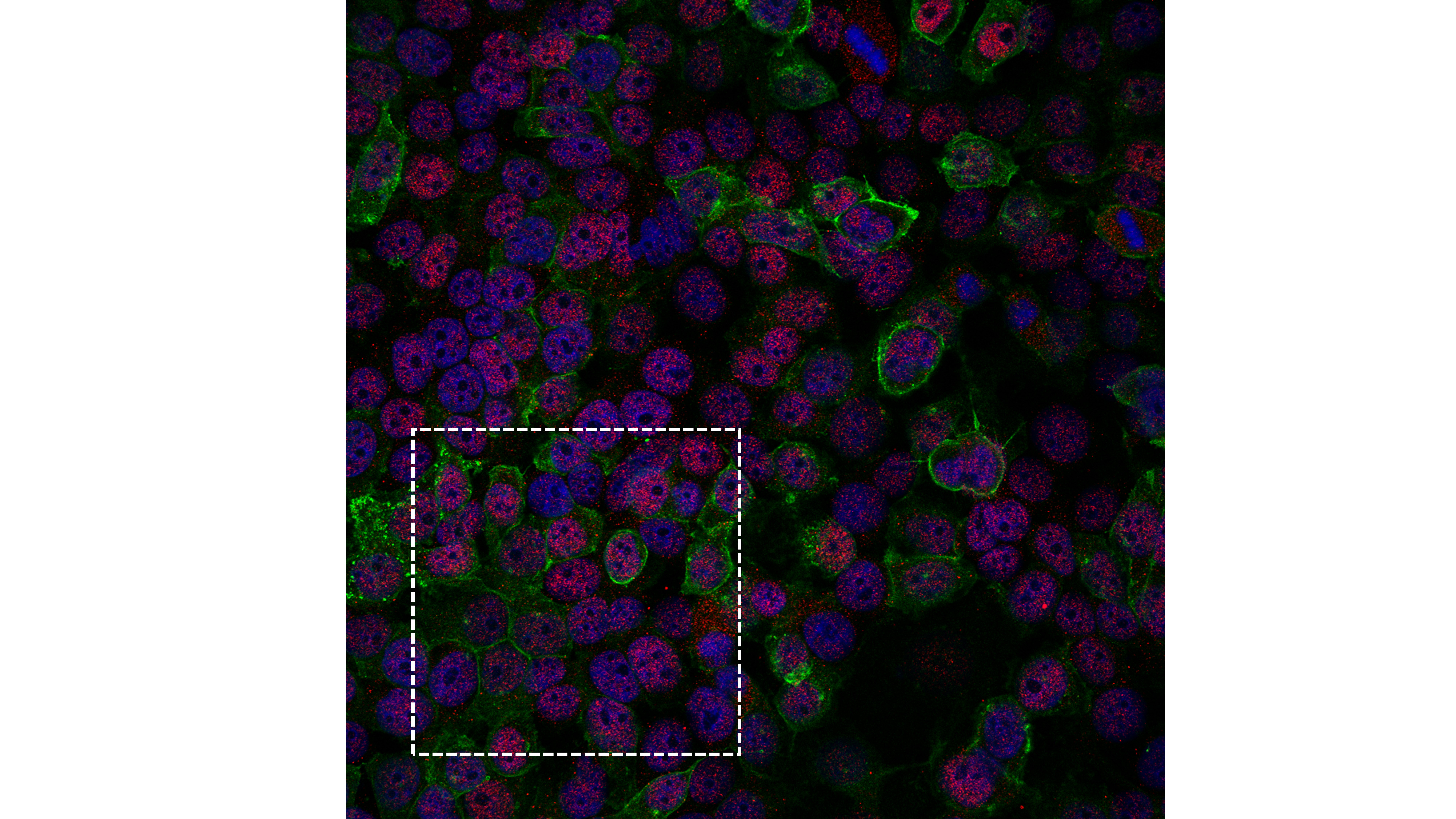

Supplement: Supplementary file 4 — Source data Fig. 1 [file 44319_2024_220_MOESM4_ESM.zip › Figure 1/FIG 1E/Fig_1E_roi/1E_MLN4924_DOX_subset.JPG]

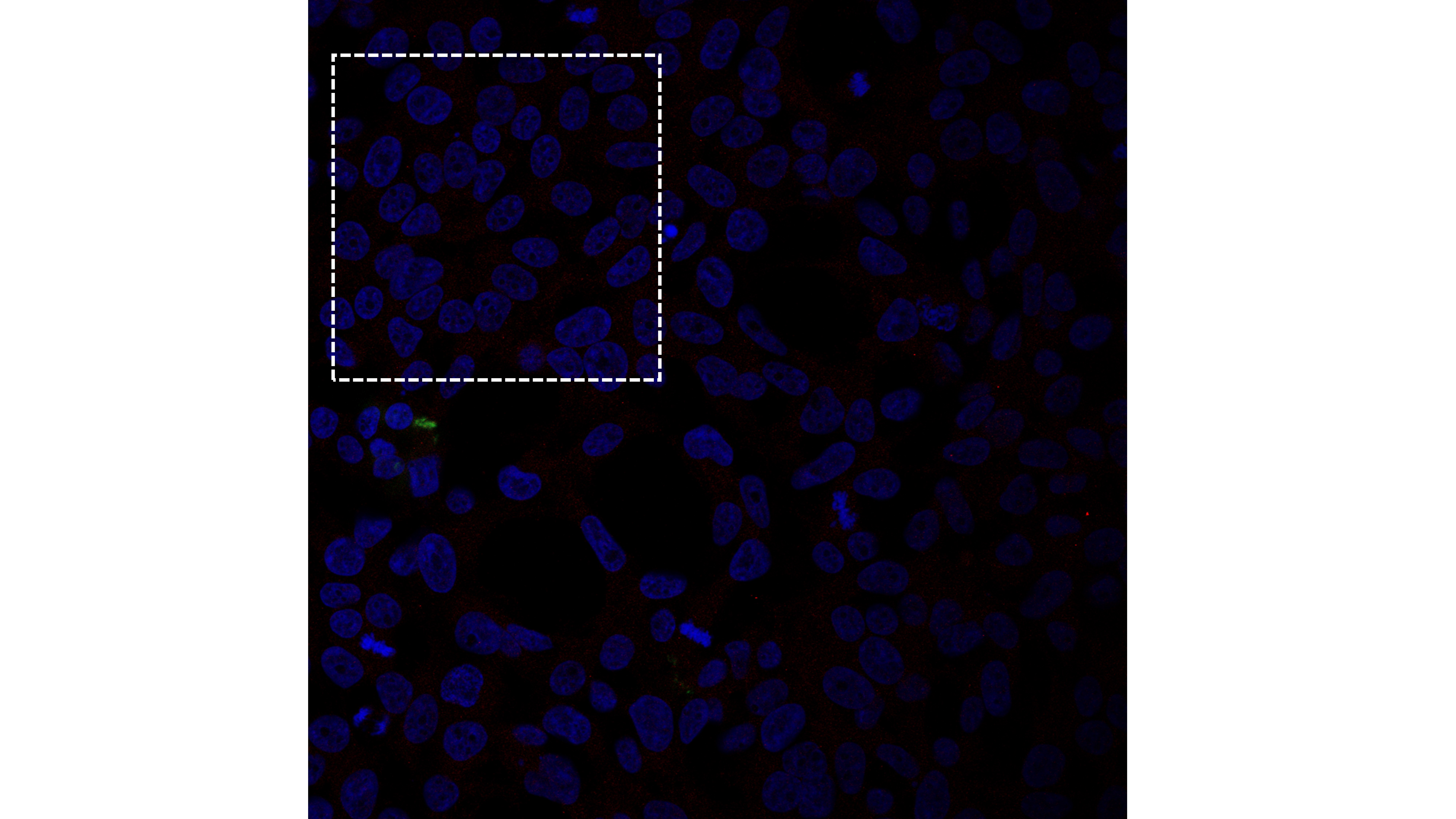

Supplement: Supplementary file 4 — Source data Fig. 1 [file 44319_2024_220_MOESM4_ESM.zip › Figure 1/FIG 1E/Fig_1E_roi/1E_NO_AB_subset.JPG]

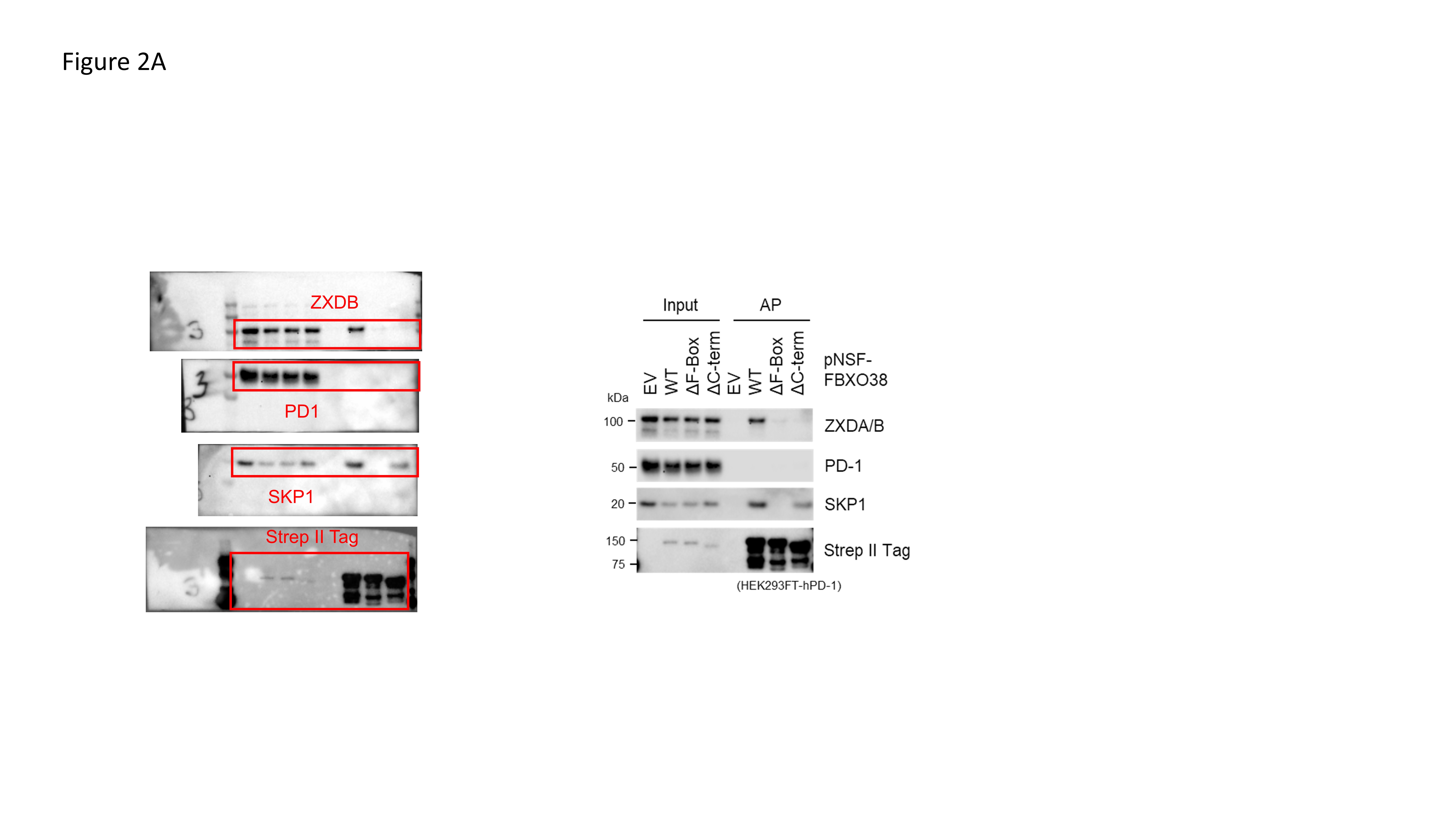

Supplement: Supplementary file 5 — Source data Fig. 2 [file 44319_2024_220_MOESM5_ESM.zip › Figure 2/2A/2A.JPG]

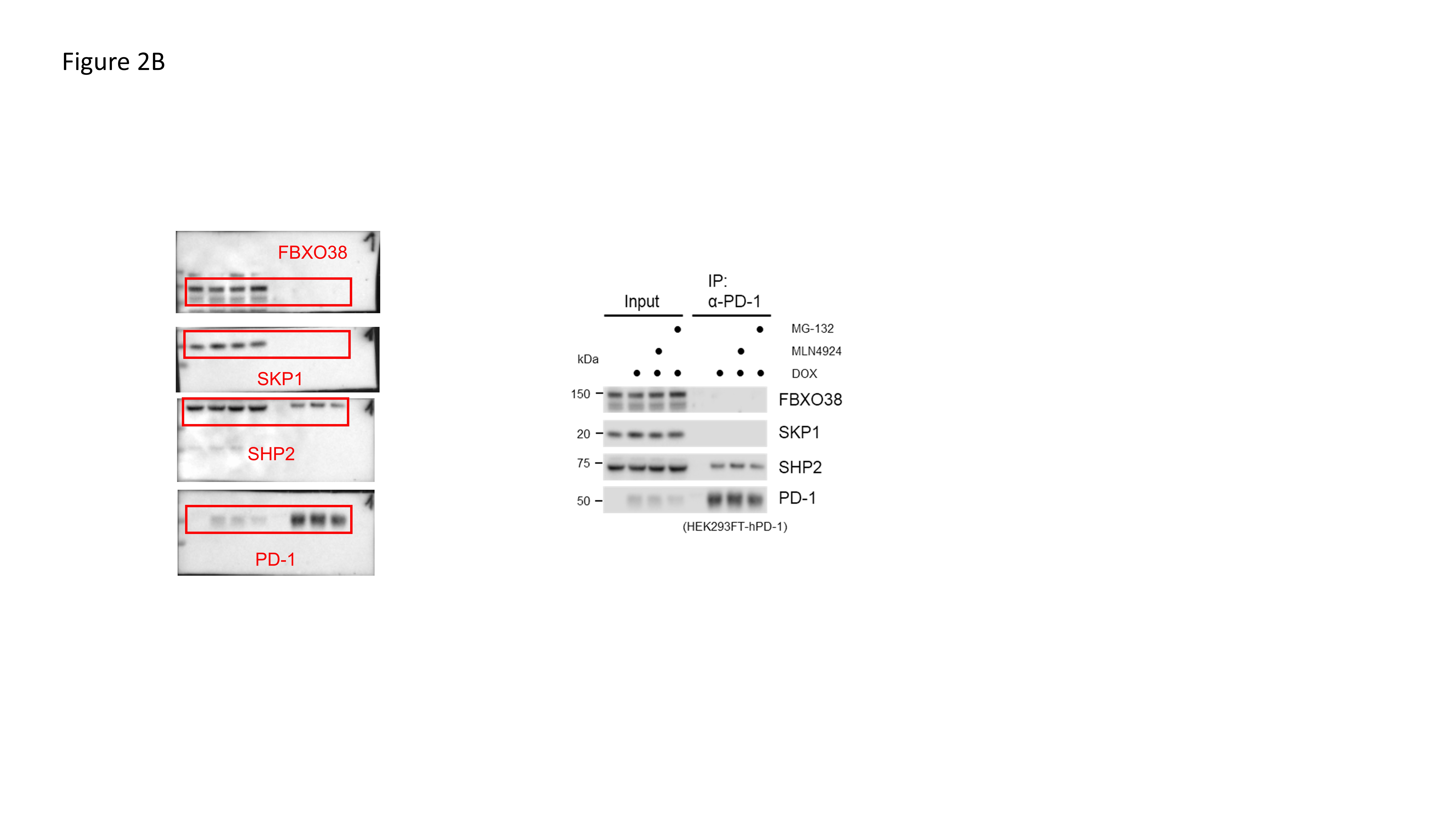

Supplement: Supplementary file 5 — Source data Fig. 2 [file 44319_2024_220_MOESM5_ESM.zip › Figure 2/2B/2B.JPG]

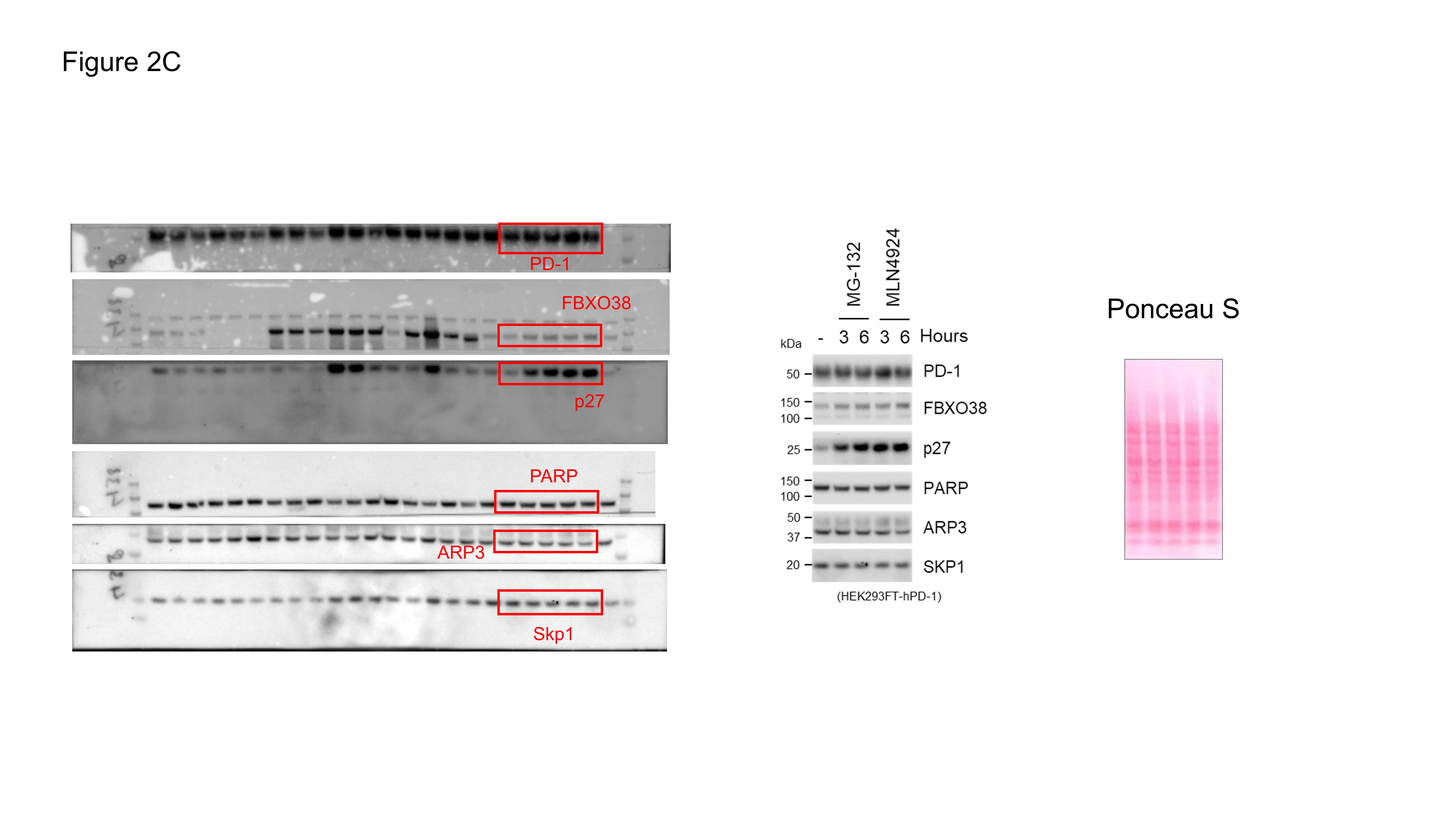

Supplement: Supplementary file 5 — Source data Fig. 2 [file 44319_2024_220_MOESM5_ESM.zip › Figure 2/2C/2C.JPG]

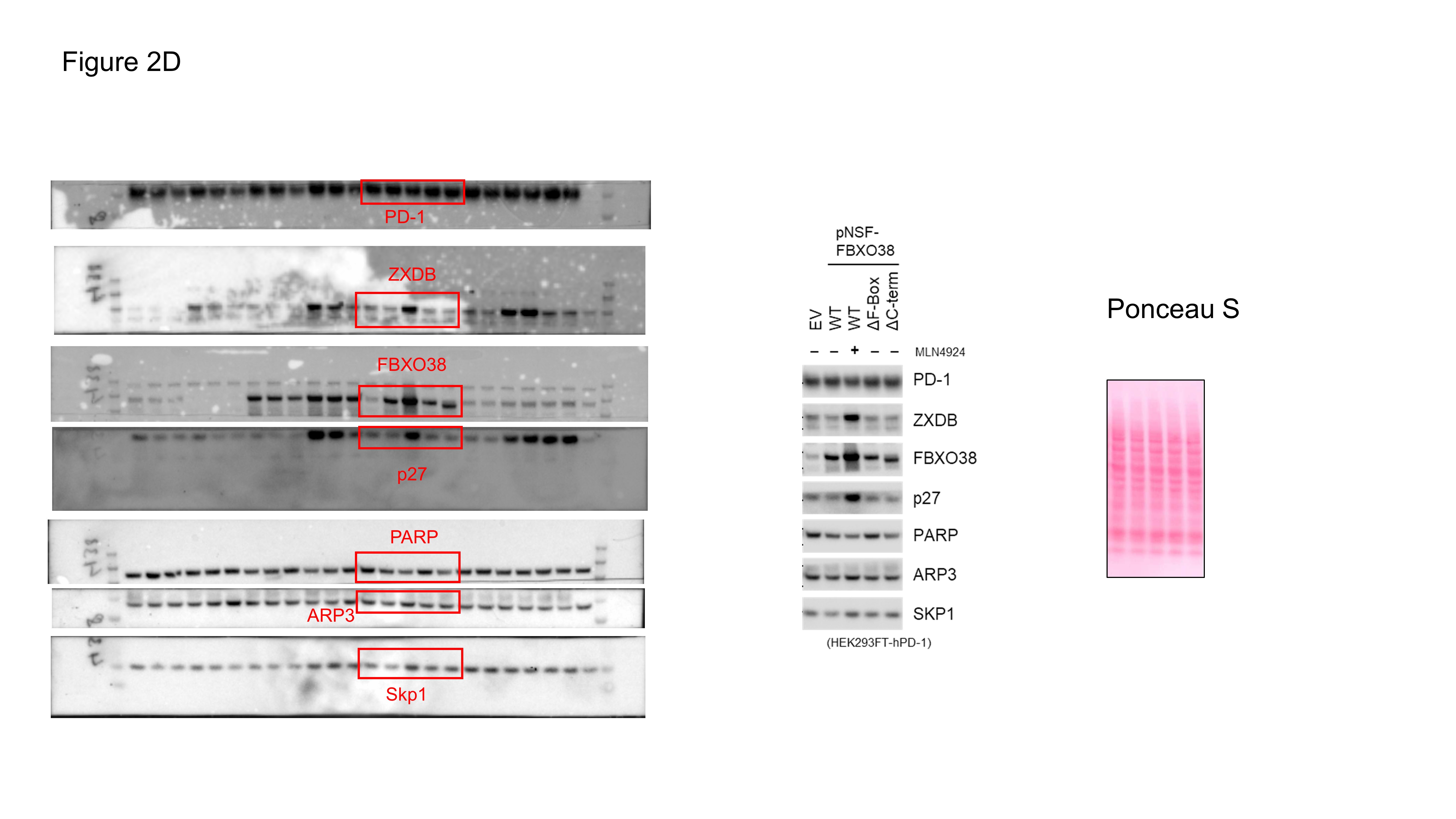

Supplement: Supplementary file 5 — Source data Fig. 2 [file 44319_2024_220_MOESM5_ESM.zip › Figure 2/2D/2D.jpg]

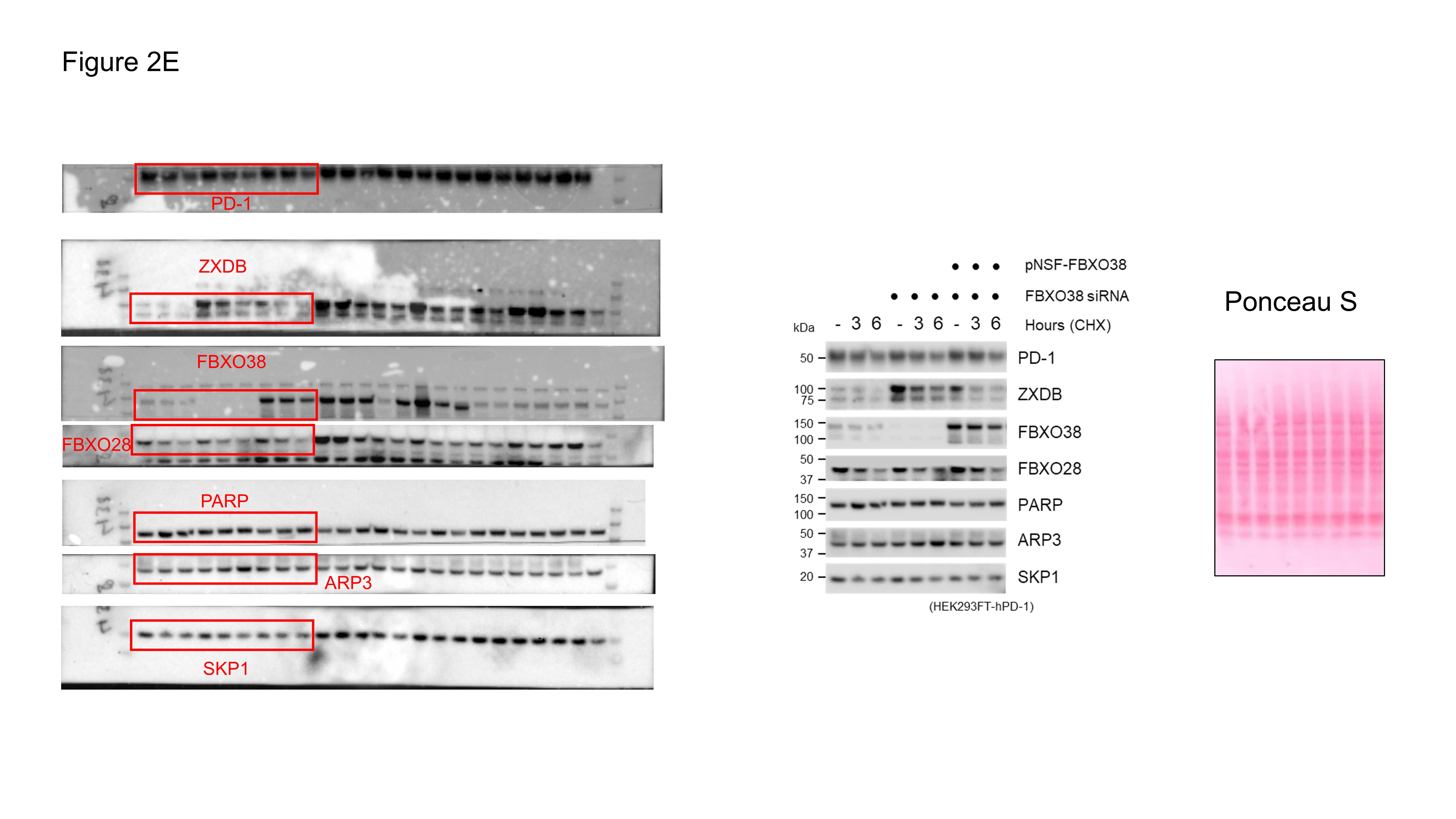

Supplement: Supplementary file 5 — Source data Fig. 2 [file 44319_2024_220_MOESM5_ESM.zip › Figure 2/2E/2E.JPG]
