# Supplementary material for: FBXO38 is dispensable for PD-1 regulation
Source: EMBO Rep. 2024 Sep 12;25(10):13. doi: 10.1038/s44319-024-00220-8 (PMC11467412; doi:10.1038/s44319-024-00220-8)
Supplement: Supplementary file 8 — Expanded View Figures [file 44319_2024_220_MOESM8_ESM.pdf]

# Expanded View Figures

**Figure EV1. FBXO38 does not control the levels and stability of PD-1 in T cells.**

(**A**, **B**) Jurkat (left) or HPB-ALL (right) T-cell leukemia cell lines were incubated for 72 h with phorbol myristyl acetate (PMA) and ionomycin (iono) followed by 6 h treatments with MG-132 or MLN4924 ($n = 3$; biological replicates). Surface PD-1 expression was analyzed by flow cytometry. Graphs represent the percentage of PD-1-positive cells (**A**) and the geometric mean of PD-1 fluorescence intensities (gMFI + SD) from all cells (**B**). (**C**) Percentage of different lymphocytic populations out of $CD45^{+}$ cells from *Fbxo38*$^{WT/WT}$ ($n = 4$; grey) and *Fbxo38*$^{KO/KO}$ ($n = 3$; red) mice. The boxes represent the 25th and 75th percentiles. Statistical significance was assessed by an unpaired two-tailed *t*-test. (**D**) Cell cycle analysis of naive or activated $CD4^{+}$ (upper panels) and $CD8^{+}$ (lower panels) T cells from *Fbxo38*$^{WT/WT}$ (WT) and *Fbxo38*$^{KO/KO}$ (KO) mice. Cells were stained with DAPI and analysed by flow cytometry. The left and middle panels present three independent samples of each genotype with the FMO control. The right panel depicts the analysis of sub-G1 (apoptosis), G1, and G2-M phases in percentage. Source data are available online for this figure.


© The Author(s)

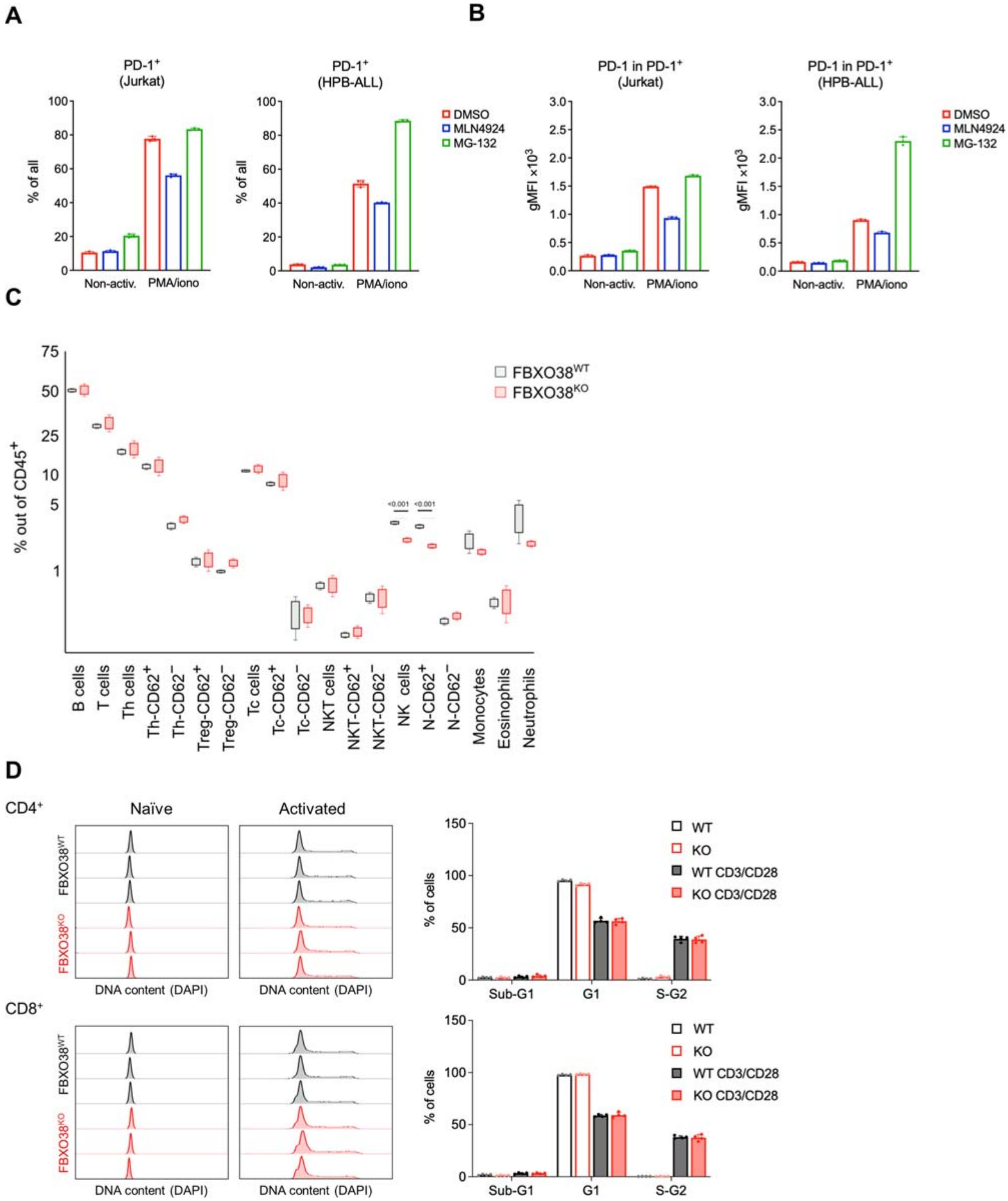
A
PD-1+ (Jurkat)
PD-1+ (HPB-ALL)
% of all
Non-activ.
PMA/iono
DMSO
MLN4924
MG-132
B
PD-1 in PD-1+ (Jurkat)
PD-1 in PD-1+ (HPB-ALL)
gMFI ×10³
C
% out of CD45+
FBXO38WT
FBXO38KO
<0.001
B cells
T cells
Th cells
Th-CD62+
Th-CD62−
Treg-CD62+
Treg-CD62−
Tc cells
Tc-CD62+
Tc-CD62−
NKT cells
NKT-CD62+
NKT-CD62−
NK cells
N-CD62+
N-CD62−
Monocytes
Eosinophils
Neutrophils
D
CD4+
CD8+
Naïve
Activated
DNA content (DAPI)
% of cells
WT
KO
WT CD3/CD28
KO CD3/CD28
Sub-G1
G1
S-G2

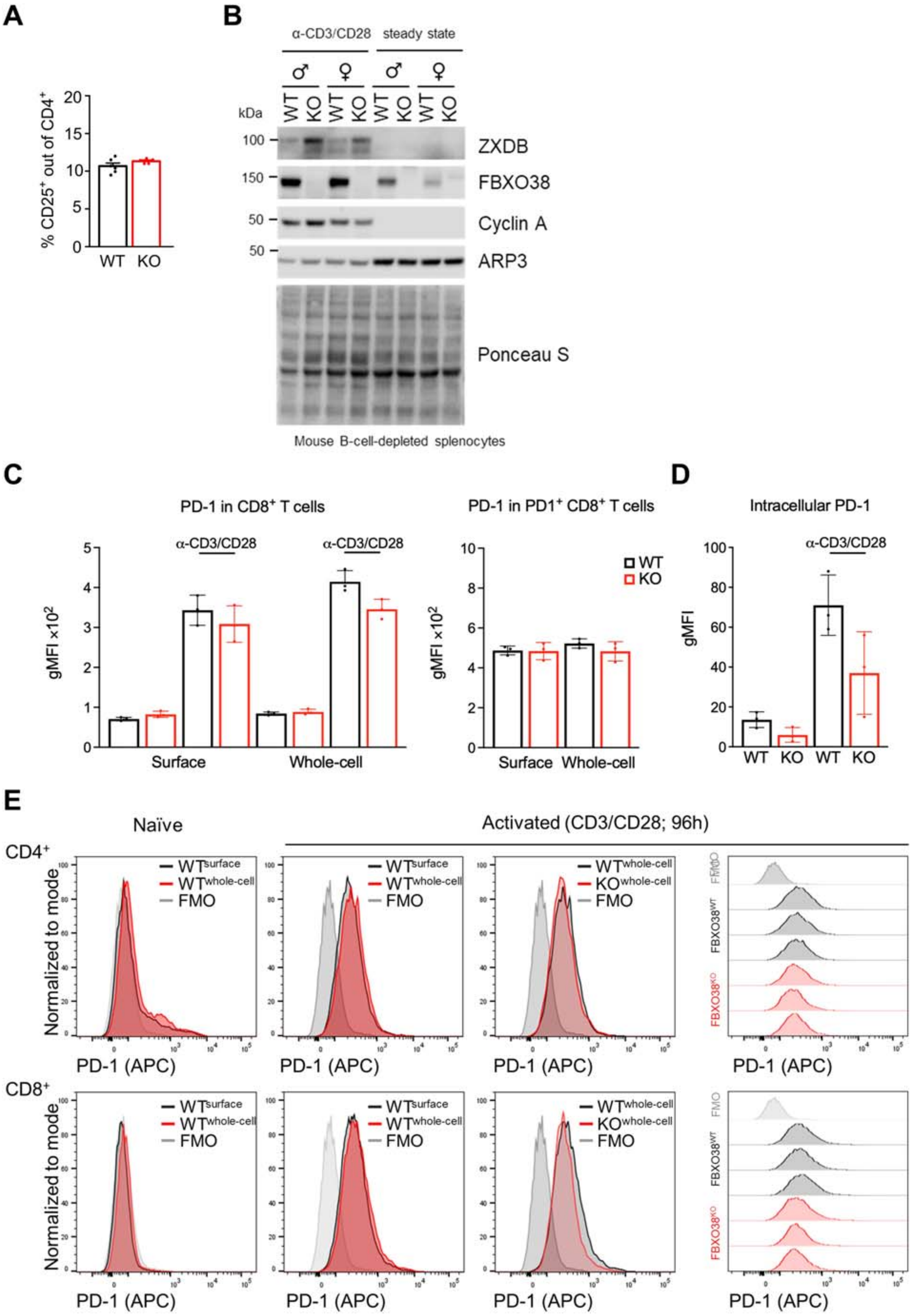
A
% CD25+ out of CD4+
WT KO
B
α-CD3/CD28 steady state
♂ ♀ ♂ ♀
WT KO WT KO WT KO WT KO
kDa
100
150
50
50
ZXDB
FBXO38
Cyclin A
ARP3
Ponceau S
Mouse B-cell-depleted splenocytes
C
PD-1 in CD8+ T cells
α-CD3/CD28
gMFI ×10²
Surface Whole-cell
PD-1 in PD1+ CD8+ T cells
WT
KO
Surface Whole-cell
D
Intracellular PD-1
gMFI
WT KO WT KO
E
Naïve
Activated (CD3/CD28; 96h)
CD4+
CD8+
Normalized to mode
WTsurface
WTwhole-cell
KOwhole-cell
FMO
FBXO38WT
FBXO38KO
PD-1 (APC)

**Figure EV2. FBXO38 does not control the levels and stability of PD-1 in T cells.**

(**A**) Percentage of $CD25^+$ cells out of $CD4^+$ T cells. The bars represent mean + SEM. $n = 6$ *Fbxo38*$^{WT/WT}$ (WT) and 5 *Fbxo38*$^{KO/KO}$ (KO) mice in two independent experiments (same as in Fig. 3H). (**B**) T cells isolated from mouse splenocytes (same as in Fig. 3H, I) were stimulated with anti-CD3/CD28 beads in presence of IL-2 for 96 h. Naive or anti-CD3/CD28 stimulated T cells were lysed and immunoblotted as indicated. ZXDB or cyclin A staining was used as the positive control and ARP3 as a loading control. Ponceau S staining was utilized to demonstrate protein loading. (**C**) Surface or whole-cell expression of PD-1 in naive or activated $CD8^+$ T cells from WT and Fbxo38 KO mice (same as in Fig. EV3D). Fixed cells were stained for surface markers (CD4 or CD8) and then either directly stained for PD-1 (surface) or alternatively permeabilized before PD-1 staining (whole-cell staining) as shown in scheme on the left panel. Samples were then analyzed by flow cytometry. Graphs represent the geometric mean of PD 1 fluorescence intensities (gMFI + SD) in $CD8^+$ T cells (left) or in PD-$1^+$ $CD8^+$ T cells (right). (**D**) Intracellular PD1 gMFI in naive or activated $CD8^+$ T cells from WT and Fbxo38 KO mice (same as in Fig. EV3G) as a result of difference between whole-cell and surface staining. (**E**) Surface or whole-cell expression of PD-1 in naive or activated $CD4^+$ (upper panel) and $CD8^+$ (lower panel) T cells from WT and Fbxo38 KO mice. Histograms show representative animal. The right panel shows three independent staining in comparison with naive cells.

© The Author(s) 

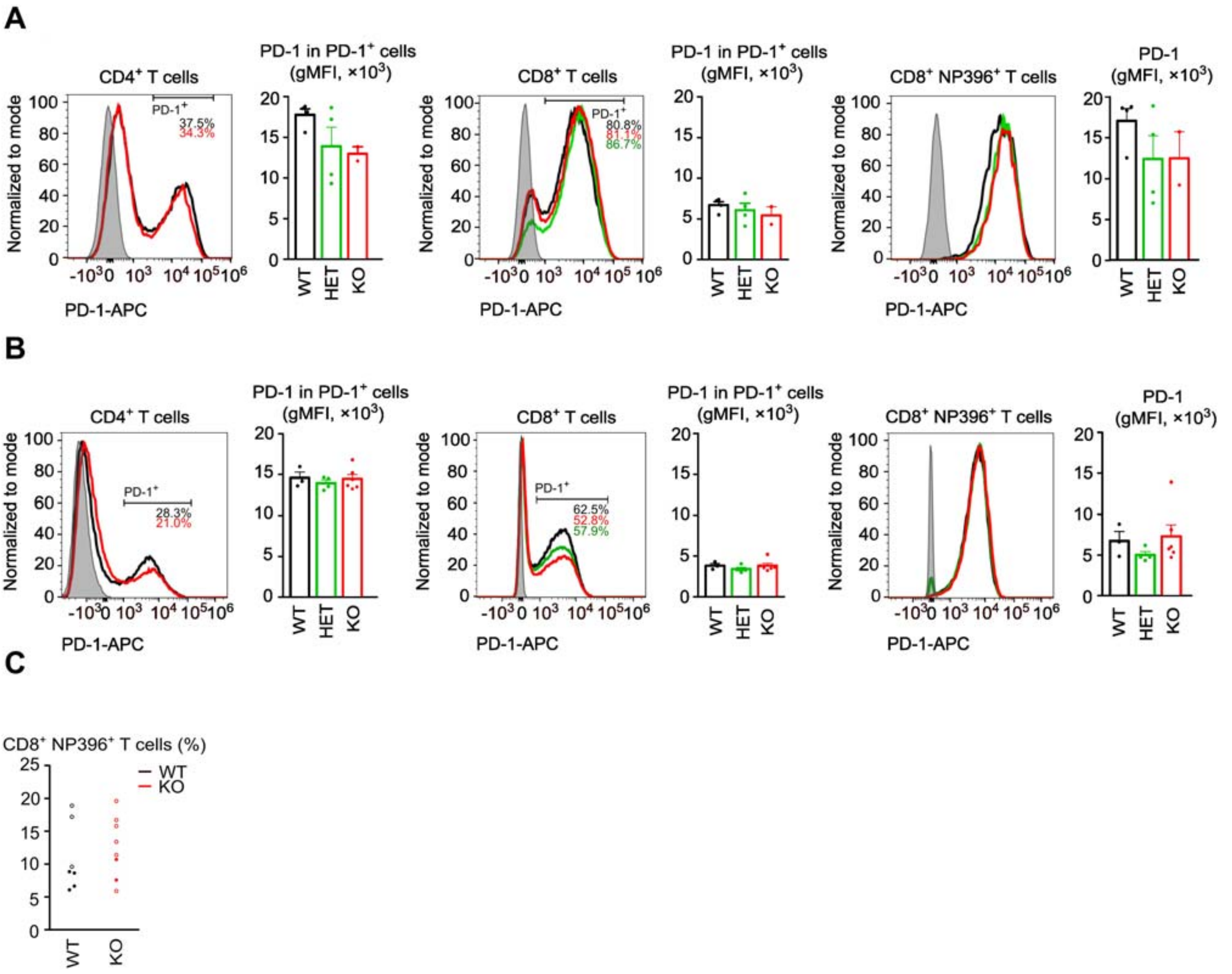


**Figure EV3. Fbxo38 does not control the levels or stability of PD-1 upon viral infection.**

(**A**, **B**) Cell-surface expressions of PD-1 in T cells isolated from the spleens of LCMV-infected *Fbxo38*$^{WT/WT}$ and *Fbxo38*$^{KO/KO}$ mice. The results of two independent experiments (**A**, **B**) are presented as normalized data in Fig. 4C. Histograms represent the analysis of representative samples. Bars show cell-surface PD-1 levels in CD4$^+$ PD-1$^+$, CD8$^+$ PD-1$^+$and CD8$^+$ D$^b$-NP396 tetramer$^+$ T cells (all PD-1$^+$ gate). $n = 7$ *Fbxo38*$^{WT/WT}$, 8 *Fbxo38*$^{KO/KO}$, and 8 *Fbxo38*$^{WT/KO}$ mice. (**C**) WT and KO mice were infected with LCMV. The percentage of Db-NP396 tetramer+ LCMV-specific T cells out of splenic CD8$^+$ T cells was quantified 8 days post-infection by flow cytometry (same experiments as shown in Fig. 4C).

 © The Author(s)
